# Diagnostic Accuracy of Lipid Transfer Proteins (LTPs) Specific IgE Assay in Food Allergy: A Systematic Review

**DOI:** 10.3390/ijms252312925

**Published:** 2024-12-01

**Authors:** Chiara Bellia, Davide Stefano Sardina, Concetta Scazzone, Domenico Lio, Letizia Scola, Carina Gabriela Uasuf

**Affiliations:** 1 Department of Biomedicine, Neurosciences and Advanced Diagnostics, University of Palermo, 90127 Palermo, Italy; chiara.bellia@unipa.it (C.B.); concetta.scazzone@unipa.it (C.S.); letizia.scola@unipa.it (L.S.); 2 Transfusion Medicine Unit, University Hospital “Paolo Giaccone”, 90127 Palermo, Italy; 3 Institute of Translational Pharmacology (IFT), National Research Council (CNR), 90146 Palermo, Italy; davidestefano.sardina@gmail.com; 4 University Research Centre “Migrate”, University of Palermo, 90100 Palermo, Italy; domenico.lio@people.unipa.it

**Keywords:** food hypersensitivity, lipid transfer protein, IgE, component-resolved diagnostics

## Abstract

The aim of this systematic review was to evaluate the diagnostic accuracy of molecular-based LTPs serum sIgE for the diagnosis of food allergies in patients with suspected allergy to one of the LTPs-containing foods. Cohort, prospective or retrospective cross-sectional studies were considered for inclusion in this review. Oral food challenge (both open and double-blind placebo-controlled) was the reference standard for the diagnosis. PubMed (MEDLINE), Web of Science, Scopus, and ClinicalTrial.org were searched for relevant papers. The risk of bias was assigned by the QUADAS-2 tool. Data were reported as the sensitivity and specificity. The study protocol was registered in the PROSPERO database (CRD42022321985). Fifteen articles, including 2395 individuals, were included. The sensitivity of Ara h 9 for peanut allergy diagnosis ranged from 6 to 61%; the specificity ranged from 57 to 100%. The Cor a 8 sensitivity ranged from 11 to 43%, with the specificity ranging from 59 to 94%. Ara h 9 and Cor a 8 serum sIgE may be useful for confirming the diagnosis of peanut and hazelnut allergy in symptomatic patients, although the diagnostic accuracy is limited by the low sensitivity. More investigation on other LTPs and in adult populations may be important to define the clinical role of this test in food allergy diagnostics.

## 1. Introduction

Food allergy (FA) is an adverse reaction to food that is mediated by the immune system. The clinical symptoms range from mild symptoms with only oral itching or dermal symptoms to systemic life-threating anaphylaxis. In the last few decades, the prevalence of food allergy has increased, and it is estimated to be about 13% in Europe [1], becoming a public health issue. Valuable insights into FA have been obtained over the last few years regarding the causes and the mechanisms of FA as well as new developments in diagnostics. In routine clinical settings, the diagnosis of FA is based on clinical presentation and in vitro specific IgE to whole-allergen extracts. The oral provocation test, the gold standard for the diagnosis of FA, is performed only by expert allergists due to its safety profile. Indeed, it is limited to cases with an inconclusive clinical pathway. The development of component-resolved diagnostics has gained much attention in the last years, specifically in poly-sensitized patients where the sensitization patterns are complex, and recognition of primary sensitization and cross-reactivity remains challenging. In the Mediterranean area, many food allergies are mainly due to lipid transfer proteins (LTPs) allergens contained in fresh fruits, nuts, wheat and vegetables.

LTPs are non-glycosylated proteins anchored to the plasma membrane. The molecular structure of LTPs is highly conserved and comprises four alpha helices stabilized by disulfide bridges with a characteristic hydrophobic internal cavity. The biological role of LTPs is extracellular transfer and deposition of various lipids to form complex barrier macromolecules on the surface of leaves, roots, and seeds [2]. LTP sensitization has not been reported in America and Africa, while it is highly prevalent in the Mediterranean area. For example, it has been demonstrated that the prevalence of Pru p 3 sensitization is about 9–12% in countries such as Italy or Spain [3,4]. Sensitization can be asymptomatic in some cases, but it can also elicit severe and systemic reactions. Overall, the variability of clinical presentation and different geographic distributions of sensitization profiles have led to significant uncertainty about the clinical reliability of in vitro LTP allergen molecular diagnostics [5]. Although the accuracy of LTP-specific IgE assay remains unclear, its use in clinical practice is increasing due to the availability of new in vitro diagnostic systems. Indeed, the diagnostic accuracy of component-resolved diagnostics has been evaluated in several meta-analysis, but few data about LTP have been reported [6,7,8]. The aim of this systematic review was to describe the sensitivity and specificity of the serum LTP-specific IgE test for the diagnosis of FA in symptomatic patients using oral food challenge as the diagnostic reference standard.

## 2. Materials and Methods

### 2.1. Reporting and Study Protocol Registration

This review was conducted and reported according to the PRISMA statement (Table S1) [9]. The study protocol was registered in the PROSPERO database before starting the literature search (CRD42022321985, available from https://www.crd.york.ac.uk/prospero/display_record.php?ID=CRD42022321985 (accessed on 30 November 2024)).

### 2.2. Eligibility Criteria

The review question was as follows: What is the diagnostic accuracy of molecular-based LTPs allergens serological tests (allergen-based serum IgE) for the diagnosis of food allergies? Due to the nature of the systematic review, namely diagnostic accuracy evaluation, we considered the Population, Index test, and Target condition (PIT) to organize our review question instead of the well-established PICO framework, which is specifically intended for intervention studies [10]. We included studies recruiting patients with symptoms of food allergies (skin and mucosa, gastrointestinal, respiratory symptoms, anaphylaxis) as the population of interest, with no specific restrictions as to the context. The index test was specific IgE antibodies against any LTP, determined using singleplex or multiplex measurement. The target condition was allergy due to LTP-containing food (apple, peach, kiwi, strawberry, pomegranate, walnut, peanut, hazelnut, almond, chestnut, tomato, celery, wheat, lettuce, lentil, corn, grape, olive). The oral provocation test, both open food challenge (OFC) or double-blind, placebo-controlled food challenge (DBPCFC), was the reference standard for food allergy diagnosis. Studies assessing the diagnostic accuracy, including cohort, prospective or retrospective cross-sectional studies, were considered. To be considered in this review, studies must include a clear definition of the target population, the target condition, the index test, and the reference standard. Case-control studies were excluded. Narrative reviews, editorials, comments, and any type of paper not presenting quantitative data on the research question were excluded.

### 2.3. Search Strategy and Information Sources

We searched PubMed (MEDLINE), Web of Science, Scopus, and ClinicalTrial.org for reports published from the database inception to the date of search. The databases were searched on 19 April 2022. The literature search was performed again on the 23 May 2024 to retrieve the most recent reports. The approach used to develop the search strategy was adopted from intervention studies considering the non-experimental setting and specifically defining population of interest, index test, and condition. The literature search was developed using the guidance for describing the search strings for systematic reviews in the form of PRISMA-S [11]. The full search strategy used for MEDLINE was strictly adapted to search the other databases (Supplementary Materials File S1). No restriction on language was applied.

### 2.4. Selection of Studies

Duplicates were identified and removed by automation tools. Initially, two reviewers (CB, CU) independently reviewed the first 20 records and discussed any inconsistency until a consensus was reached. Then, two co-authors proceeded with screening the remaining records based on the title and abstract and working independently (CB, CU, LS, CS). The screening was conducted using Rayyan software (https://www.rayyan.ai/) [12]. No automation tools were used for study exclusion. Disagreement was resolved by the consensus of the reviewers. Full texts of the records that passed the first selection were screened by two co-authors working independently (CB, CU, LS, CS) to ensure that inclusion criteria were fulfilled. In each step of the records screening, the reviewer was blinded to the decision of the other one.

### 2.5. Data Collection Process

Two reviewers extracted data independently into a standardized Excel format, including any relevant information about the study design, sample size, selection criteria, demographics, symptoms, culprit food, manufacturer of laboratory reagents, cut-off for test positivity, type of OFC (CB, CU, LS, CS, DSS). The form was piloted and calibration exercises on five records were conducted prior to the formal data extraction to ensure consistency between reviewers. Disagreement between collectors was resolved by consensus.

### 2.6. Quality Assessment

The risk of bias (ROB) and applicability of the included studies were evaluated by the QUADAS-2 (Quality Assessment of Diagnostic Accuracy Studies) tool [13]. Briefly, it was conducted in four phases: (a) reporting the review question; (b) developing review-specific guidance for quality assessment and writing a template for the ROB assessment for each primary study; (c) reviewing a flow diagram of the primary study or constructing one if it was not available; and (d) evaluating the risk of bias and applicability for each item. Regarding the review-specific tailoring, the question “Were the index test results interpreted without knowledge of the results of the reference standard?” was omitted from the ROB assessment template because the index test was a quantitative and objective measure, so no subjective interpretation was requested for positive/negative classification. Regarding the question “Is the reference standard likely to correctly classify the target condition?”, the target condition was defined with the highest grade of certainty by double-blind placebo-controlled (DBPC) food challenge [14], so the answer to the signaling question was “yes” only if DBPC challenge was considered as a reference standard, and “no” if open food challenge was used for diagnosis. For the signaling question “Was there an appropriate interval between index tests and reference standard?”, the authors considered that the appropriate interval between the index test and the reference standard was within one year; if the two tests were performed within the diagnostic work-up, the answer was “yes”. As suggested by the QUADAS-2 group, the ROB was considered “low” if the answers to all the signaling questions were “yes”, and “high” if at least one signaling question was answered “no”. Two reviewers (CB, CU, LS, CS) independently assessed the risk of bias for the primary studies using the review-specific template constructed in accordance with the QUADAS-2 tool. Any disagreement was resolved through discussion.

### 2.7. Data Synthesis, Analysis and Reporting

The diagnostic accuracy was described by the sensitivity and specificity calculated from the two-by-two table for each study. The sensitivity and specificity for each study were reported by forest plots. Initially, only studies evaluating the diagnostic accuracy of Ara h 9 and Cor a 8 were considered for quantitative analysis because too few studies reported data about other LTPs (Pru p 3) to be included in the quantitative analysis. The random effect, hierarchical summary receiver operating characteristic (HS-ROC) model did not converge, so a narrative synthesis of the results was reported. The heterogeneity between studies was investigated by visually inspecting the forest plots of the sensitivity and specificity. The studies were grouped according to the age of participants, use of DBPC vs. oral food challenge, and different cut-offs. Data analysis was performed using MetaDTA v2.1.2 [15].

## 3. Results

### 3.1. Study Characteristics and Risk of Bias

**Figure 1:**
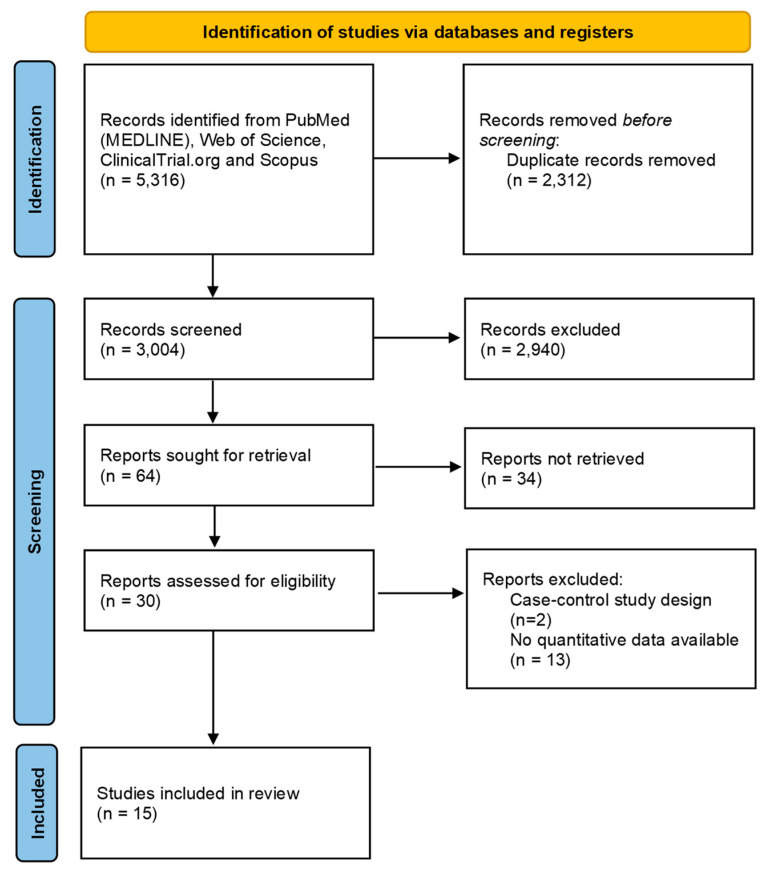
PRISMA flow chart reporting the numbers of records screened, assessed for eligibility, and included in this review.

After removing duplicates, 3004 abstracts were screened for inclusion. Then, 64 full-text articles were assessed for eligibility. We excluded 34 of these, leaving 30 publications to be included. Fifteen articles were further excluded because no quantitative data were available (n = 13) or for the wrong study design (n = 2). Finally, 15 articles were included in the review [16,17,18,19,20,21,22,23,24,25,26,27,28,29,30]. The selection process is described in Figure 1.

All the included studies had a cross-sectional design. Overall, this systematic review includes 2395 subjects, among whom 1312 had an allergy to peanut (nine studies), 149 to peach (two studies), and 934 to hazelnut (three studies). Most patients were pediatric, with only three studies including adult patients (age ranging from 18 to 46 years). In most cases, the patients had only local symptoms, but in six studies patients with anaphylaxis were also included. In only five studies, all the patients underwent OFC; in the others, the percentage of participants who had undergone OFC was quite variable, ranging from 15% to 98%. In these cases, the reason for not performing OFC was a history of anaphylaxis after the ingestion of the culprit food or denying informed consent. Component-resolved diagnostics was performed for all the participants in most of the studies, except for four studies in which the test was available for 16–98% of participants. Singleplex ImmunoCAP was used in most of the studies, except for two, in which multiplex assay was used. In five studies, the researchers received funding from the manufacturers of laboratory reagents. The main study characteristics are summarized in Table 1.

**Figure 2:**
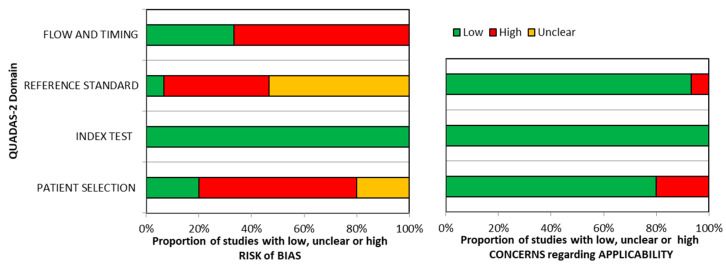
Proportion of studies with a low, high or unclear risk of bias and concerns regarding applicability according to the QUADAS-2 domains.

The quality assessment of the included studies is summarized in Table 2 and Figure 2.

No risk of bias was detected for the domain “index test”. Similarly, there were no applicability concerns for this domain. Few commercial sIgE assays are available at present and the threshold is pre-specified in almost all the studies, so there is a high grade of concordance between the included studies and the review question on this domain. The “Reference standard” domain was the mostly affected by the risk of bias, probably because most of the included studies used an open challenge to diagnose food allergy instead of DBPCFC, which is considered the gold standard for food allergy diagnosis. In 10 studies, only a variable fraction of the enrolled patients (15–98%) underwent oral food challenge, affecting the risk of bias for the domain “patient selection”. Concerns regarding applicability were high in a small proportion of studies (<10%), probably because the review question was not specific about the diagnostic criteria for food allergy, so the results from most studies could be applied.

### 3.2. Peanut

**Figure 3:**
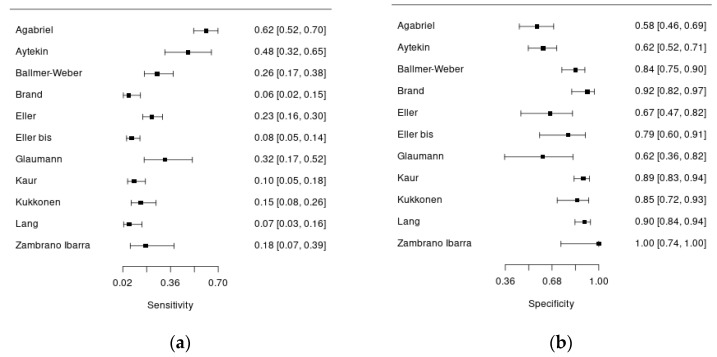
Forest plots of the Ara h 9 sensitivity (**a**) and specificity (**b**) for the diagnosis of peanut allergy.

Overall, 10 studies assessed the diagnostic accuracy of Ara h 9, using 0.35 kU/L (seven studies) and/or 0.1 kU/L (four studies) cut-offs. All the studies had a cross-sectional design and were conducted in Europe, except for the study by Kaur et al. that was conducted in Australia and the one by Aytekin et al. in Africa. Figure 3a,b show forest plots summarizing the sensitivity and specificity of Ara h 9 for the diagnosis of peanut allergy.

Overall, 1312 patients with suspected peanut allergy were included in this analysis, and in 806 of them, the diagnosis of food allergy was confirmed. Anaphylaxis ranged from 0 to 24% in the included studies. In almost all the studies, pediatric patients were enrolled, except for the study by Ballmer-Weber et al., who recruited adult patients (mean age 26 years). The percentage of males ranged from 40% to 68%. There was significant heterogeneity regarding the reference standard used to diagnose peanut allergy: six studies adopted an open food challenge while four studies adopted the double-blind placebo-controlled one. Moreover, the percentage of patients undergoing food challenge was quite variable, ranging from 15 to 100%. Regarding the index test, all the studies used an ImmunoCAP assay (Thermo Fisher) for serum anti-Ara h 9-IgE measurement, in singleplex or multiplex, except for two studies. The sensitivity of Ara h 9 for peanut allergy diagnosis was quite low, ranging from 6 to 61%, with acceptable specificity, ranging from 57 to 100%.

### 3.3. Hazelnut

**Figure 4:**
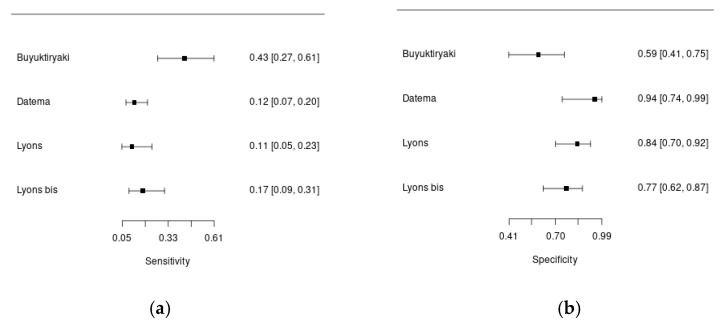
Forest plots of the Cor a 8 sensitivity (**a**) and specificity (**b**) for the diagnosis of hazelnut allergy.

Three studies enrolled patients with suspected hazelnut allergy. Figure 4a,b show forest plots summarizing the sensitivity and specificity of Cor a 8 for the diagnosis of hazelnut allergy in the included studies.

Overall, 934 patients were included in this analysis, with 171 diagnoses of hazelnut allergy. One study included children; the others included adult patients (mean age ≅ 33 years). The percentage of males ranged from 29% to 70%. In all the studies, DBPCFC was used to diagnose food allergy, but the percentage of patients undergoing the challenge was variable (17–87%). The cut-off was always pre-specified. In the study by Lyons et al., the authors assessed the diagnostic accuracy of component-resolved diagnostics using both 0.1 kU/L and 0.35 kU/L cut-offs, and they found that in both cases, the specificity of Cor a 8 for hazelnut allergy was high, but the overall diagnostic accuracy was poor [25]. The sensitivity of Cor a 8 for the diagnosis of hazelnut allergy was low, ranging from 11 to 43%, with higher specificity, ranging from 59 to 94%.

### 3.4. Peach

Although the role of Pru p 3 in the diagnosis of peach allergy has been evaluated in many studies, only two studies fulfilled the inclusion criteria for this systematic review [18,29] In both studies, DBPCFC was conducted by administering peeled peach pulp to patients selected due to having a clear history of hypersensitivity reactions after contact with or ingestion of peach, with the aim of excluding the effect of Pru p 3, mainly expressed in the peach peel and considered as a confounder for the clinical diagnostic pathway. For this reason, the results arising from these studies should be considered with caution in relation to Pru p 3 sensitization.

## 4. Discussion

This systematic review describes the diagnostic accuracy of serum specific IgE against LTPs in food allergy. Overall, most of the evidence concerns peanut, hazelnut and peach allergy. The main findings of this analysis are that the LTP-specific IgE assay has very low sensitivity and acceptable specificity, making this test suitable for confirming food allergy when food challenge, considered the gold standard for food allergy diagnosis, is not practicable or not informative. For this reason, the results confirm the role of this test as an add-on in the clinical pathway of food allergy in adjunction with standard diagnostic procedures, including the skin prick test, and oral food challenge.

During the last few years, clinical laboratory methods in allergology have experienced technological improvement, leading to the release of new assays for serum IgE that are specific for molecular allergenic components. This revolution, based on increasing knowledge of the biochemical properties of allergens and on their geographic distribution, is enhancing the comprehension of IgE-mediated allergies toward precision medicine. Until today, hundreds of molecular allergens have been identified, isolated, and characterized, and it is expected that this number will increase rapidly. In allergic reactions, alongside species-specific molecules, ubiquitous molecules could be involved too. These molecules are expressed in many botanical or animal species. Inevitably, IgE sensitization to these components, often referred to as panallergens, can evoke varying degrees of cross-reactivity. LTPs can be considered panallergens because they are expressed in several pollens and plant foods belonging to both the widespread Rosaceae family and many other distantly related species and can elicit systemic reactions in LTP-sensitized subjects. Often, the primary source of LTP (Pru p 3)-sensitization is peach, which arises from epidemiological studies mainly from the Mediterranean area, such as Italy, Spain, and Greece [31]. Nevertheless, the clinical relevance of LTP allergens, as well as the primary sensitizer, varies greatly depending on the patient’s age and geographic area [2]. Many studies have described the diagnostic accuracy of component-resolved diagnostics, but no one has evaluated the specific involvement of sIgE against LTPs allergens. This topic is of particular interest, especially in countries of the Mediterranean area, where LTP-driven allergies are more frequent. In the study by Nilsson et al., the diagnostic accuracy of Ara h 9 was evaluated using both 0.10 kU/L and 0.35 kU/L cutoffs. The authors found a pooled specificity ranging from 0.77 to 0.81 and a pooled sensitivity ranging from 0.32 to 0.19, based on the cut-offs [7]. Similarly, Flore Kim et al. found that the Ara h 9 assay had a diagnostic sensitivity of 0.14 and a specificity of 0.85 at the 0.35 kU/L cut-off [6]. The present analysis confirms these previous findings, demonstrating that LTPs component-resolved diagnostics is suitable when confirmation of a peanut allergy diagnosis is required, but it should be used with caution for excluding the disease. Indeed, the high specificity limits the false positives; on the other hand, the suboptimal sensitivity means that a high number of false negatives may occur. Overall, this information may help clinicians in the diagnostic process leading to a food allergy diagnosis. With respect to Cor a 8 and the diagnosis of hazelnut allergy, we found a sensitivity ranging from 11 to 43% and a specificity ranging from 59 to 94%. In the study by Masthoff et al., the sensitivity of Cor a 8 was quite low, 0.06, and there was acceptable specificity, 0.96 [32]. Again, we can confirm that Cor a 8 is a suitable test when the diagnosis of hazelnut allergy confirmation is expected, but the test may be misleading for the exclusion of the disease.

The main limit of this systematic review is that the risk of bias was high in many studies for the domain “reference standard” due to the use of different challenge protocols (open versus double-blind food challenge) and the different proportions of patients undergoing the reference standard protocol. In this regard, it should be noted that for ethical reasons, patients with severe systemic symptoms, such as anaphylaxis, or with a clear diagnosis of FA must be excluded from life-threating or unnecessary diagnostic tests, such as oral food challenge. Nevertheless, in the review protocol, oral food challenge was considered the gold standard for the diagnosis of food allergy, so a large number of studies that did not meet the review’s inclusion criteria, namely the ones that did not consider OFC as the definitive test for diagnosis, were excluded. On the other hand, no limits for the studies’ geographical area were considered in the present review protocol.

In conclusion, this review suggests that some serum LTP-specific IgE assays, such as Ara h 9 and Cor a 8, could be useful in the diagnostic pathway of peanut and hazelnut allergies with high specificity but low sensitivity. Nevertheless, further studies with an adequate study design, avoiding the case-control one, and employing DBPCFC as a reference standard are needed to define the diagnostic accuracy of sIgE against LTP assay. Moreover, the role of these tests in the diagnosis of food allergy in adults is quite unexplored so requires more investigations.

## Figures and Tables

**Table 1:** Main characteristics of the studies included in this systematic review.

Study		Participants					Reference Standard		Index Test			
Author	Selection Criteria	Symptoms	N	Age, Mean or Min–Max	Male, %	Culprit Food	Participants Who Underwent OFC, %	Food Challenge	Participants Who Underwent CRD, %	LTP	Index Test	Cut-Off
Agabriel et al. 2014 [16]	History of peanut allergy. Exclusion criteria: CRD not performed	Peanut-induced symptoms (not specified)	181	6.3	68%	Peanut	15%	Open	100%	Ara h 9	ImmunoCAP, Singleplex	0.1 kU/L
Aytekin et al. 2023 [28]	Suspected peanut allergy	Peanut-induced symptoms (not specified)	123	1–18	74%	Peanut	NA	Open	100%	Ara h 9	ALEX2, multiplex	0.3 kU/L
Ballmer-Weber et al. 2015 [17]	Positive DBPCFC; history of anaphylaxis.	Skin and mucosal 33%; gastrointestinal 6%; respiratory 0.6%; anaphylaxis 26%	150	26	40%	Peanut	57%	DBPC	100%	Ara h 9	ImmunoCAP, Singleplex	0.35 kU/L
Boyano-Martínez et al. 2013 [18]	History of immediate hypersensitivity reactions.	Skin and mucosal 95%; gastrointestinal 18%; Respiratory 21%	57	7.4	56%	Peach pulp	98%	Open (objective signs) DBPC (subjective signs)	100%	Pru p 3	ImmunoCAP, Singleplex	0.35 kU/L
Brand et al. 2021 [19]	Atopic pediatric patients with Sensitization to peanut; OFC.	Skin 82%; asthma 49%; rhinitis 57%	117	6.9	73%	Peanut	100%	DBPC	100%	Ara h 9	ISAC	0.35 ISU
Buyuktiryaki et al. 2016 [20]	(i) History of early phase reaction after hazelnut ingestion; (ii) hazelnut sIgE ≥ 0.35 kU/L; (iii) positive SPT	Skin 87.3%; respiratory 36.5%, anaphylaxis 12%	64	3.4	70%	Hazelnut	87%	DBPC	86%	Cor a 8	ImmunoCAP, Singleplex	0.1 kU/L
Datema et al. 2015 [21]	Immediate adverse reactions occurring 2 h after the ingestion of hazelnuts	Oral mucosal 84.4%; respiratory and gastrointestinal ranged from 20.7 to 35.4%	731	32.3	37%	Hazelnut	17%	DBPC	16%	Cor a 8	ImmunoCAP, Singleplex	0.35 kU/L
Eller et al. 2023 [27]	Suspected peanut allergy due to clinical history; and/or positive SPT; and/or positive IgE against peanut extract	Suspected peanut allergy (symptoms not specified)	157	5.6	NA	Peanut	100%	NA	100%	Ara h 9	ImmunoCAP, Singleplex	0.1 kU/L
Glaumann et al. 2012 [22]	Suspected peanut allergy with positive IgE or SPT. Exclusion criteria: (i) antihistaminic or steroids prior to food challenge; (ii) history of anaphylaxis	Suspected peanut allergy (symptoms not specified)	43	12	55%	Peanut	88%	DBPC	100%	Ara h 9	ImmunoCAP, Singleplex	0.1 kU/L
Kaur et al. 2021 [23]	Previous reaction to peanut or sensitization	Skin 41%; asthma 41%; rhinitis 49%; anaphylaxis 14%	222	8	66%	Peanut	100%	Open	100%	Ara h 9	ImmunoCAP, Singleplex	0.35 kU/L
Kukkonen et al. 2015 [24]	(i) History of a moderate-to-severe allergic reaction at peanut exposure; (ii) sensitization, any grade. Exclusion criteria: (i) poor asthma control; (ii) any major chronic illness	Skin 45%; asthma 53%	102	9	56%	Peanut	100%	DBPC	100%	Ara h 9	ImmunoCAP, Singleplex	0.35 kU/L
Lang et al. 2022 [30]	Children undergoing OFC with at least one peanut component testing result	Skin 50%; asthma 19%; rhinitis 34%	184	4	60.3%	Peanut	100%	Open	100%	Ara h 9	ImmunoCAP, Singleplex	0.35 kU/L
Lyons et al. 2022 [25]	Suspected hazelnut allergy undergoing food challenge	Skin 58%; asthma 53%; rhinitis 89%; anaphylaxis 21%	139	33	29%	Hazelnut	100% (in 36% it was inconclusive)	DBPC	88%	Cor a 8	ImmunoCAP, Singleplex	0.1 kU/L and 0.35 kU/L
Vílchez-Sánchez et al. 2023 [29]	History of immediate hypersensitivity reactions related to intake or contact with peach	Contact urticaria 42%; skin and mucosal 91%; generalized urticaria 30%; respiratory 11%; gastrointestinal 12%; anaphylaxis 29%	92	10	53%	Peach	46%	NA	98%	Pru p 3	ImmunoCAP, Singleplex	0.35 kU/L
Zambrano Ibarra et al. 2019 [26]	Clinical history of IgE-mediated reaction to peanuts or nuts	Skin 36%; respiratory 67%; anaphylaxis 24%	33	8	66%	Peanut	NA	Open	100%	Ara h 9	ImmunoCAP, Singleplex	0.1 kU/L

Selection criteria include the inclusion criteria; exclusion criteria are specified only if they were reported by the authors in the primary study. CRD: component-resolved diagnostics; DBPC: double-blind placebo-controlled; FEIA: fluorescence enzyme immunoassay; ISAC: immuno-solid phase allergen chip; SPT: skin prick test; NA: not available; IQR: interquartile range.

**Table 2:** Risk of bias assessment and applicability concerns for each study included in this review. Risk of bias was classified as high (red), low (green) or unclear (yellow).

Study	RISK OF BIAS				APPLICABILITY		
	PATIENT SEL.	INDEX TEST	REF. STAND.	FLOW AND TIMING	PATIENT SEL.	INDEX TEST	REF. STAND.
Agabriel et al. 2014 [16]	High	Low	High	High	High	Low	Low
Aytekin et al. 2023 [28]	High	Low	High	High	Low	Low	Low
Ballmer-Weber et al. 2015 [17]	High	Low	Low	High	High	Low	Low
Boyano-Martínez et al. 2013 [18]	Unclear	Low	High	High	Low	Low	Low
Brand 2021 [19]	High	Low	Unclear	High	Low	Low	Low
Buyuktiryaki 2016 [20]	High	Low	Unclear	High	Low	Low	High
Datema 2015 [21]	High	Low	Unclear	Low	Low	Low	Low
Eller 2023 [27]	High	Low	Unclear	High	Low	Low	low
Glaumann 2012 [22]	Unclear	Low	Unclear	High	High	Low	Low
Kaur 2021 [23]	low	Low	High	Low	Low	Low	Low
Kukkonen 2015 [24]	low	low	Unclear	Low	Low	Low	Low
Lang 2022 [30]	Unclear	Low	High	Low	Low	Low	low
Lyons 2022 [25]	Low	Low	Unclear	High	Low	Low	Low
Vílchez-Sánchez 2023 [29]	high	low	Unclear	high	Low	low	low
Zambrano Ibarra 2019 [26]	High	Low	High	Low	Low	Low	Low

## Data Availability

Dataset available on request from the authors.

## Supplementary Materials

The following supporting information can be downloaded at https://www.mdpi.com/article/10.3390/ijms252312925/s1.
